# Caspases help to spread the message via extracellular vesicles

**DOI:** 10.1111/febs.16418

**Published:** 2022-03-18

**Authors:** Claire Hill, Elizabeth R. Dellar, Luis Alberto Baena‐Lopez

**Affiliations:** ^1^ 6396 Sir William Dunn School of Pathology University of Oxford UK; ^2^ 6396 Nuffield Department of Clinical Neurosciences University of Oxford UK

**Keywords:** apoptosis, caspases, EVs, extracellular vesicles, intercellular communication, non‐apoptotic

## Abstract

Cell‐cell communication is an essential aspect of multicellular life, key for coordinating cell proliferation, growth, and death in response to environmental changes. Whilst caspases are well‐known for facilitating apoptotic and pyroptotic cell death, several recent investigations are uncovering new roles for these enzymes in biological scenarios requiring long‐range intercellular signalling mediated by extracellular vesicles (EVs). EVs are small membrane‐bound nanoparticles released from cells that may carry and deliver cargo between distant cells, thus helping to coordinate their behaviour. Intriguingly, there is emerging evidence indicating a key contribution of caspases in the biogenesis of EVs, the selection of their cargo content, and EV uptake/function in recipient cells. Here, we discuss the latest findings supporting the interplay between caspases and EVs, and the biological relevance of this molecular convergence for cellular signalling, principally in non‐apoptotic scenarios.

## Graphical abstract

Caspase enzymes have been attributed both apoptotic and non‐apoptotic functions in the modulation of cell communication via extracellular vesicles (EVs). Caspase cleavage facilitates actin remodelling and subsequent membrane blebbing for EV formation. Moreover, caspase‐cleavage liberates proteins and RNA, regulating their EV incorporation and thus the messages delivered to recipient cells. In some instances, caspases themselves are directly EV‐loaded, facilitating cargo cleavage within EVs, or activating signalling mechanisms in recipient cells.

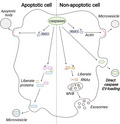

**Abbreviations**
ABs: apoptotic bodies
EVs: extracellular vesicles
ILVs: intraluminal vesicles
MVBs: multivesicular bodies
MVs: microvesicles

## Introduction

Cells must adjust their intracellular homeostasis and molecular activities in response to a plethora of dynamic environmental factors. In multicellular organisms, coordinated cellular behaviour is critical to ensure proper organ morphogenesis and physiology [1]. Cell behaviour is harmonised via precise signalling mechanisms facilitating the exchange of “information” (signalling molecules and cellular contents) between cells in close proximity and far away. This sophisticated intercellular dialogue allows many cellular processes (e.g. cell proliferation, growth, death, and migration) and physiological events (e.g. mounting an effective immune response) to occur. Various different forms of signalling exist, involving direct cell contact [juxtacrine, for example, filopodia, cytonemes, microtubule (MT)‐nanotubes and tunnelling nanotubes (TNTs) [2, 3, 4, 5]] or secreted factors; mainly soluble proteins (e.g. cytokines) and chemical small molecules (e.g. reactive oxygen species, nitric oxide, metabolites) [6, 7, 8, 9]. The latter can influence the activity of both nearby cells (e.g. paracrine signalling) and cells that are far apart (e.g. hormone transport through the bloodstream; endocrine signalling) [10]. Extracellular vesicles (EVs) play a prominent role as part of both these short‐ and long‐distance forms of signalling between cells. EVs are a highly heterogenous group of membrane‐bound carriers which are released by all types of cells, across species, that sustain a wide range of biological functions in physiological and pathological conditions [11, 12, 13, 14, 15, 16]. EVs have been implicated in mediating non‐targeted damage to surrounding cells after exposure to cellular stressors such as radiation and cytotoxic drugs, known as the bystander effect [17, 18, 19, 20, 21, 22], whilst other studies have proposed that they act primarily as proliferative or anti‐apoptotic factors [23, 24, 25, 26, 27]. There is growing evidence indicating a regulatory role for the evolutionarily conserved caspase family in the bystander effect [22, 25, 28] and many aspects of EV biology (e.g., cargo loading, release, and uptake/processing of EVs in target cells) [29, 30, 31, 32, 33, 34, 35, 36, 37, 38, 39, 40, 41, 42, 43, 44, 45].

Caspase enzymes are a family of cysteine aspartic proteases, traditionally considered facilitators of cell death through the genetically regulated programs of apoptosis or pyroptosis [46]. However, more recent evidence in a range of human and model systems has attributed novel roles to these enzymes beyond cell death, such as the regulation of stem cell function and cell migration [47, 48, 49, 50, 51, 52, 53, 54, 55, 56], with this review exploring functions for these enzymes in modulating EV biology, particularly in non‐apoptotic contexts. The involvement of EV‐mediated cell signalling and disruption of caspase activity have separately been shown to be clinically relevant, and often pave the way to diseases such as cancer [57, 58] or neurodegeneration [13, 59, 60]. Given the functional overlap of caspase signalling and EV‐mediated cell communication, there is now a great deal of interest in exploring the molecular mechanisms underlying their intersection in both lethal and non‐lethal scenarios. In this review, we will provide a basic overview of EV biology and the evidence suggesting the participation of caspase enzymes in the molecular mechanisms mediating EV biogenesis, cargo loading or processing, and uptake.

## Overview of extracellular vesicles

### EV classification and biogenesis

Extracellular vesicles are often classified into sub‐types based on their biogenesis pathway, size and origin, principally exosomes (40–100 nm), microvesicles (MVs; 100–1000 nm) and apoptotic bodies (ABs; 50–5000 nm) [61, 62]. Exosomes derive from the endosomal system, whereby invagination of the endosomal membrane generates intraluminal vesicles (ILVs), thus forming the multivesicular body (MVB). MVBs may be trafficked to the lysosome where their fusion permits degradation of contents or protein recycling to other cellular locations [63]. Alternatively, however, MVBs may be transported to the plasma membrane where fusion allows the release of ILVs into the extracellular environment, forming exosomes [64]. Biogenesis of exosomes is thought to rely on two major pathways, the first of which is the ESCRT‐dependent process. The ESCRT machinery is made up of four main complexes (ESCRT‐0, ‐I, ‐II and ‐III) and associated proteins, such as vacuolar protein sorting‐associated protein 4 (Vps4), ALG‐2 interacting protein X (ALIX) and syntenin‐1, many of which are key protein markers associated with EVs [65, 66]. Alongside its importance in cellular processes such as cytokinesis, viral release and neuronal pruning, the ESCRT machinery is also responsible for sorting of ubiquitinated proteins into MVBs [67, 68]. However, exosomes can also be formed via ESCRT‐independent processes in which tetraspanins such as CD9, CD81 and CD63, canonical EV markers, are critical [69, 70]. These proteins are a superfamily of small four transmembrane proteins that regulate protein loading and EV formation in endosomal compartments. ILV formation for exosome secretion may also be induced by self‐association of ceramide in the cellular membranes to induce curvature and budding, a sphingolipid produced by the activity of neutral sphinogomyelinase 2 (nSMAse2) on sphingomyelin [71]. Transport of the MVB to the plasma membrane requires interaction and movement through the microtubule network facilitated by Rab small GTPases and tethering SNARE proteins (such as VAMP7, YKT6 and syntaxin 1A) [72].

Microvesicles, also known as ectosomes, originate from the plasma membrane via direct outward budding. A key factor regulating MV budding is the lipid composition of the inner and outer leaflets of the membrane bilayer. The aminophospholipids phosphatidylserine and phosphatidyl‐ethanolamine are normally located on the inner cytosolic‐facing leaflet of the cell plasma membrane, with this localisation maintained via the ATP‐dependent activity of the flippase enzyme, aminophospholipid translocase [73]. An increase of cytosolic calcium inhibits the function of the flippases, while activating additional floppases and scramblases [73]. The combined activity of floppases and scramblases results in outward lipid movement and thus their exposure in the extracellular‐facing leaflet. The coordination of these events with the calpain‐dependent remodelling of the cytoskeleton drive membrane curvature and vesicle formation prior to MV release [74, 75]. However, MV formation may also be triggered by acid sphingomyelinase‐mediated generation of ceramide, via the ionotropic ATP receptor P2X_7_, or by the action of the small GTPase ADP‐ribosylation factors ARF6 and ARF1 and their ability to phosphorylate myosin light‐chain kinase (MLCK) [76, 77, 78]. Finally, it has been shown that some of the ESCRT components are involved in both, MV and exosome biogenesis [72, 79]. For example, TSG101 may interact with ARDDC1 in a viral buddling‐like process to form MVs [80].

### EV function and cargo

Early EV studies during the 1980s revealed a role of EVs in the disposal of cellular waste, which for a long time was considered the primary function of these secreted nanoparticles [81, 82]. However, in 1996, Raposo et al. demonstrated that EVs from Epstein‐Barr virus‐transformed B lymphocytes played roles in antigen presentation and induction of a T‐cell response, hinting at a wider signalling role between producing and receiving cells [83]. Exploration of biological cargoes of EVs from the early 2000s demonstrated that their contents (protein, RNA and lipid) show some selectively in loading and that this cargo‐specificity determines their activity in recipient cells [65, 84, 85]. However, the action of EVs on recipient cells may occur by various modalities. Firstly, direct surface‐to‐surface interactions between EVs and the recipient cell surface can induce signalling cascades or facilitate the transfer of membrane constituents [83, 86, 87]. Secondly, EVs may act as delivery vehicles for bioactive molecules, by fusing to the recipient cell membrane or by endocytic uptake [88]. Delivery of both protein and RNA cargo has been demonstrated, with evidence also suggesting that some mRNAs can be translated and functionally active in recipient cells [84, 89, 90, 91, 92, 93].

Extracellular vesicles contain membrane proteins within their lipid bilayer, as well as an array of cytosolic proteins within the vesicular lumen [94, 95]. Alongside the characteristic tetraspanin markers (CD63, CD9 and CD81) [66], components of the ESCRT machinery (such as Hrs, Vps28) are commonly found. A lack of ability to physically separate EVs of distinct biogenesis pathways has hindered efforts to define EV markers distinct for each pathway. However, Mathieu et al. [96] have found that MVB‐derived EVs contain CD63 in combination with either CD9 or CD81 in HeLa cells, whilst Jeppesen et al. suggest that Annexin A1 can be used as a marker of plasma‐membrane derived MVs [97]. Several studies have also suggested that EVs are particularly enriched in RNA‐binding proteins which may have important implications for understanding EV cargo loading [98, 99].

Extracellular vesicles were first shown to contain mRNA in 2006 [100, 101], but now it is clear that other RNA species are also present (e.g. short RNAs such as miRNA, tRNA, vault RNA, snRNA and snoRNA, and long RNAs such as ribosomal RNA and long non‐coding RNAs) [102, 103, 104, 105]. However, there is currently little understanding of which species are present in different sub‐types of EVs. A key question in the EV field is how both protein and RNA cargo is distributed across the population. Barman et al. demonstrated that within “small EVs” only 10% of the total EVs (the denser fraction) contained 90% of the RNA content [106]. Importantly, observations of this kind suggest the intriguing possibility that different biogenesis pathways are used to generate specific and functionally varied types of EVs.

### EVs and cellular stress

In parallel to the investigations to characterise the defining features of distinct EV types, a substantial amount of research effort has been devoted to understanding how exposure to different cellular stimuli affects EV biogenesis, cargo and function. Multiple studies have shown an increase in the production of EVs in response to endogenous or exogenous stressors such as X‐rays [19, 107], Y‐rays [17, 108], photodynamic chemotherapy [109], heat stress [110], hypoxia [111, 112], oxidative stress [26, 110] and endoplasmic reticulum (ER) stress [113]. Beyond the variations in the number of secreted EVs, several studies have also reported changes in EV size, with particles smaller in response to heat [22] and hypoxia [27] and larger in response to ER stress [113]. However, no differences were seen in this regard in response to X‐rays [19], Y‐rays [108], hypoxia, TNF‐alpha, high glucose or mannose [114]. One can speculate that these changes in EV size may reflect altered pathways of EV biogenesis; for example, the smaller EVs seen in response to heat and hypoxia may represent an increase in the production of MVB‐derived exosomes. Under glutamine deprivation, an increase in Rab11a‐positive EVs was also seen, suggested to be indicative of EV release from recycling endosomes, rather than late endosomes, and thus reflective of altered biogenesis routes under stress [115].

Changes to EV cargo in response to cellular stress have also been widely studied, and is likely linked to changes in biogenesis, but this interaction has not yet been studied in detail. Nevertheless, changes to protein [107, 114, 116], mRNA [23, 26, 107, 114, 116] and microRNA [20, 107, 117] have been observed under different stress conditions. Several studies have also suggested, through ultraviolet or RNase treatment to inactivate the RNA, that these RNA cargo alterations are specifically responsible for mediating functional effects on recipient cells [18, 23].

Finally, evidence also suggests that EV uptake dynamics may be altered under stress conditions. Assessment of the uptake of EVs by U87MG glioma cells by labelling with the lipid dye PKH26 showed that, at 24 h after EV application, EVs derived from X‐ray irradiated cells had higher uptake than EVs from non‐irradiated controls [107]. The same increased uptake of EVs derived from irradiated cells was seen in oral squamous cell carcinoma BHY cells [108]. Intriguingly, this latter study also showed that the irradiation of recipient cells, in parallel to EV‐producing cells, significantly increased the uptake of EVs derived from either irradiated or non‐irradiated cells. These findings have suggested that the health state of both donor and recipient cells strongly determine EV biogenesis and uptake under stress conditions. Given caspases are key to the stress response, research has now focused on understanding their role in EV biology.

## Influence of caspases on EV formation

### Caspases and EV biogenesis in cell death scenarios

**Fig. 1:**
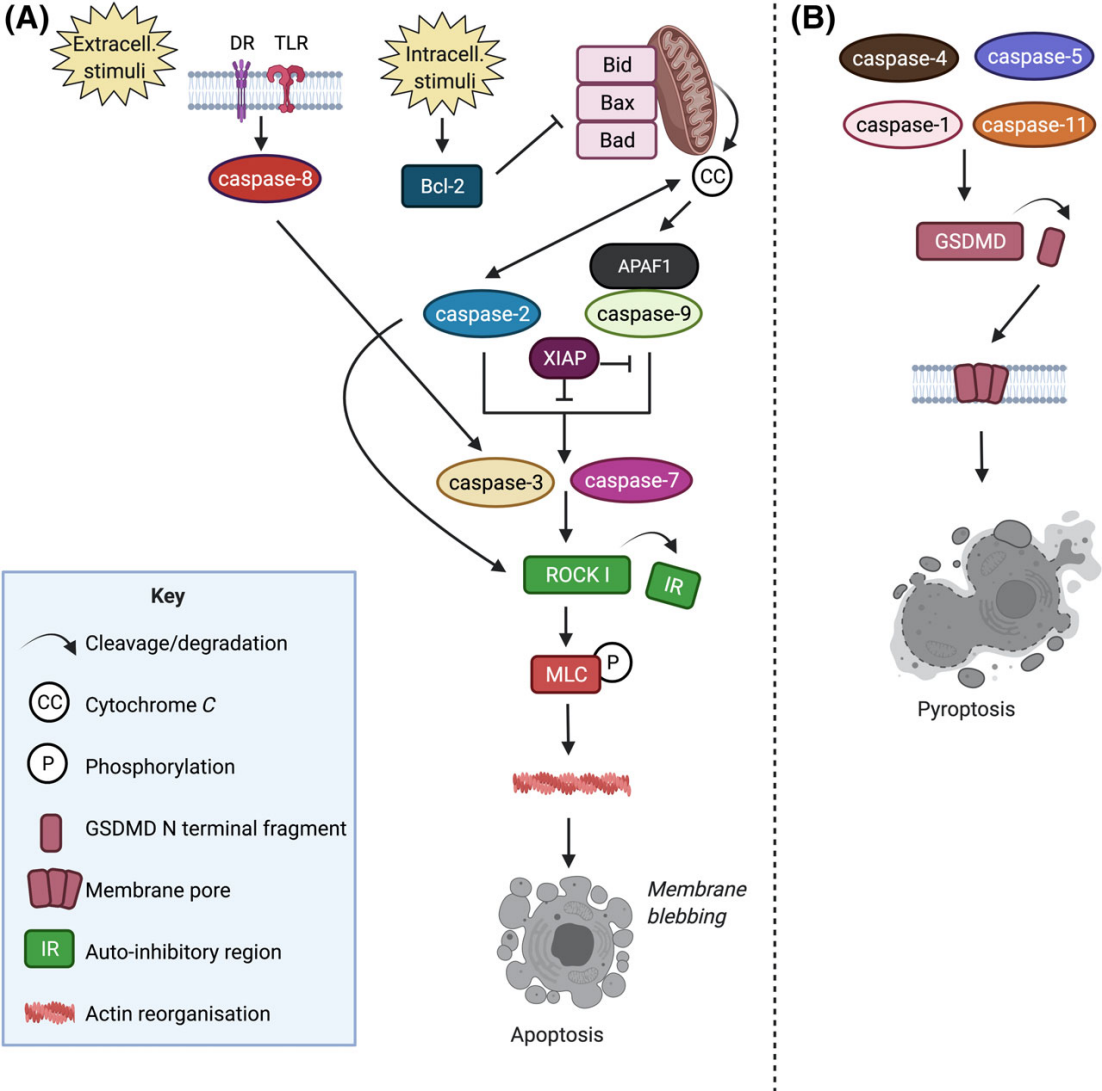
Schematic summary of caspase activity implicated in apoptotic (A) and pyroptotic (B) cell death. (A) In apoptosis, the caspase cascade, via caspase‐8, can be stimulated extracellularly via membrane death receptors (DR) or TLR. Alternatively, the caspase‐2/‐9 pathway can become stimulated via intracellular stimuli, via Bcl‐2. Activation of Bcl‐2 results in the inhibition of Bid, Bad and Bax, which results in cytochrome *C* release from mitochondria. Cytochrome *C* accumulation stimulates caspase‐2/caspase‐9 to activate caspase‐3/caspase‐7 via the aid of the adaptor protein, apoptotic protease‐activating factor‐1 (APAF1). Together APAF1 and caspase‐9 form the apoptosome. X‐linked inhibitor of apoptosis (XIAP) is an inhibitor which can prevent caspase‐9 activity, if caspase‐3/‐7 do become active, XIAP can also use an alternative domain to inhibit these caspase enzymes. Activated caspase‐3/caspase‐7 can cleave ROCK I, releasing the autoinhibitory region as a fragment. More recently, caspase‐2 has also been shown to cleave ROCK I directly. ROCK I is then able to facilitate the phosphorylation of MLC, ultimately resulting in actin reorganisation, membrane blebbing and apoptotic body release during apoptosis. (B) The inflammatory caspases (caspase‐1/‐4/‐5/‐11) cleave GSDMD, releasing an N‐terminal fragment which interacts with plasma membrane phospholipids to form pores that induce cell death via pyroptosis.

The fate of a stressed cell, towards survival or death, depends on its ability to handle the molecular consequences of that stress [118]. If a cell is unable to induce a sufficient protective response to recover from such stress, cell death may be induced by one of several cell‐death programs [46]. Apoptosis is perhaps the best‐characterised form of cell death, tightly controlled by the caspase enzymes (Fig. 1A). Apoptotic cells undergo a range of morphological changes, such as nuclear and cytoplasmic condensation, with the subsequent breaking up of the cell [119]. Similarly to MV biogenesis, caspase inactivation of flippases, and activation of scramblases initiate membrane budding, whilst caspase‐3, ‐7 and ‐2 can cleave Rho‐associated protein kinase (ROCK I) to remove an autoinhibitory region, resulting in myosin light chain (MLC) phosphorylation, actin and microtubule remodelling at the plasma membrane, and membrane blebbing, ultimately resulting in the formation of a particular subtype of EVs called ABs [120, 121, 122]. Alternatively, membrane blebbing can be facilitated by activation of ROCK II via granzymes, a family of serine proteases [123, 124]. These membrane‐bound fragments cover a range of sizes, typically large (1000–5000 nm diameter), but also smaller apoptotic vesicles (apoEVs) of 50–1000 nm diameter, comparable in size to EVs formed under normal conditions [62]. ABs and EVs have important signalling roles, including Toll‐like receptor (TLR) activation [125], suppression of proliferation [126] and pro or anti‐apoptotic functions [127, 128, 129, 130] in recipient cells. Large ABs are thought to be more distinct from other EV types, for example, being the only species to contain full‐length ribosomal RNA [131, 132], but the overlap of smaller apoEVs with EVs produced in non‐apoptotic conditions is poorly understood.

In addition to their role in apoptosis, the caspase cascade is a key component of the innate immune response, providing a first line of defence after pathogen infection. Detection of pathogen molecules or endogenous danger signals within a cell trigger the formation of the inflammasome, a multiprotein complex which promotes activation of inflammatory caspases (caspase‐1, ‐4, ‐5 and ‐11) [133, 134]. Cleavage of gasdermin D (GSDMD) by these inflammatory caspases releases an N‐terminal fragment which interacts with plasma membrane phospholipids to form pores that induce cell death via pyroptosis [135, 136, 137, 138, 139, 140] (Fig. 1B). Cleavage of interleukin‐1ß and interleukin‐18 (IL‐1 ß and IL‐18) then results in the production of proinflammatory cytokines which are released via these pores [141, 142]. Both small and large EVs have been demonstrated to be produced under such pyroptotic, as well as necrotic, conditions, but their functional significance as intercellular communicators is yet to be studied in detail [143, 144]. Beyond the inflammatory caspases, additional caspase members have been implicated in immune regulation in both mammalian and non‐mammalian organisms, via mechanisms that are incompletely understood [145, 146, 147, 148, 149, 150]. In *Caenorhabditis elegans*, Weaver et al. determined that CED‐3 (equivalent to mammalian caspase‐9) limited PMK‐1 (p38 MAPK) signalling in a non‐apoptotic manner to provide a balance point between development and the stress‐response [150]. Intriguingly, PMK‐1 has also previously been identified as a regulator of EV biogenesis in *C. elegans* [151]. Together, these findings illustrate the role of caspases as mediators of signalling in the context of immunity and provide potential insights into how the formation of EVs as a result of caspase activation can alter the cellular microenvironment.

### Caspases and EV biogenesis in non‐lethal scenarios

**Fig. 2:**
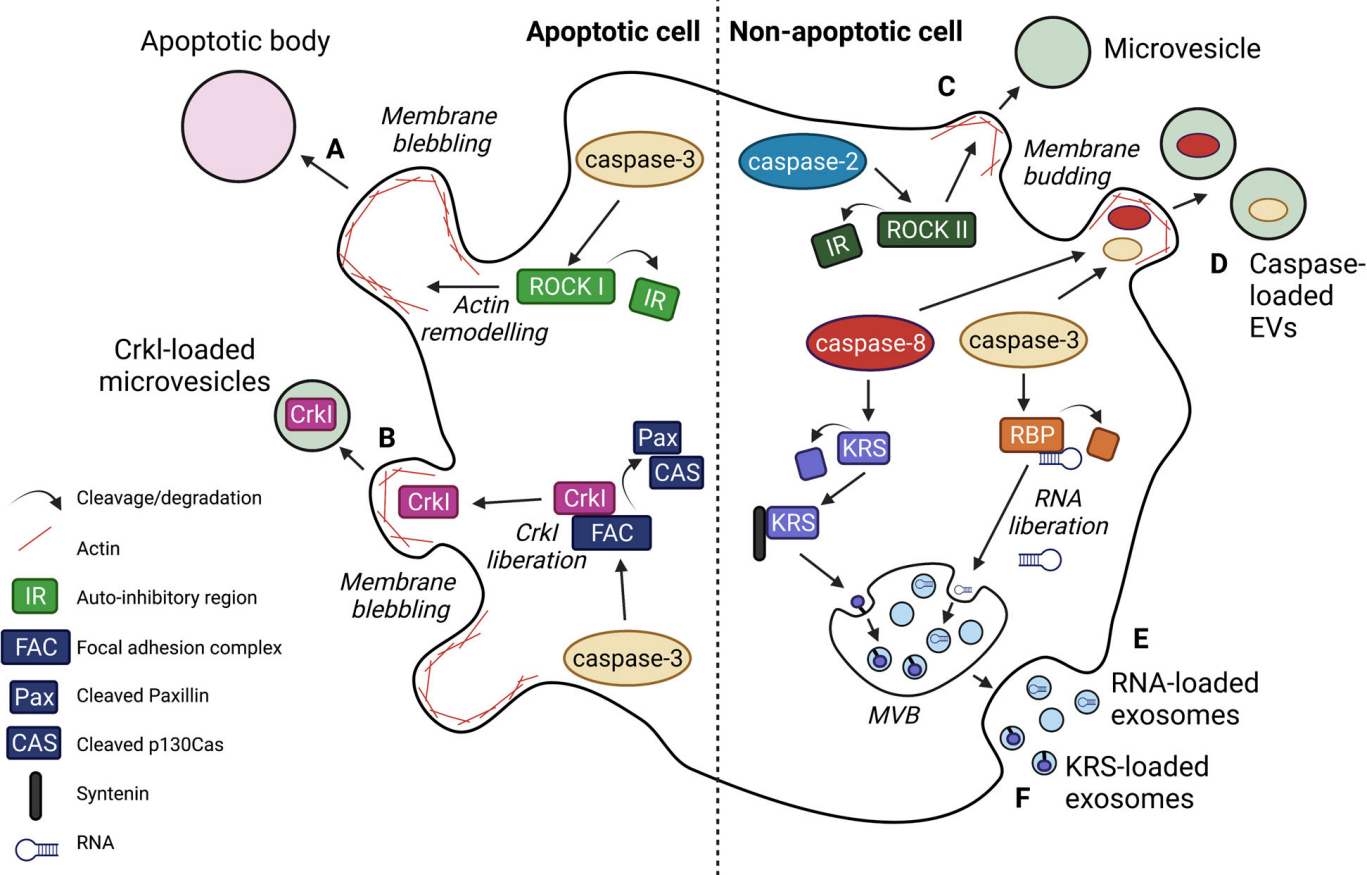
Biological examples in which caspase activity regulates EV formation and EV cargo loading, in either apoptotic or non‐apoptotic cells. (A) Caspase‐3 (also caspase‐7/‐2) cleave ROCK I in apoptotic scenarios to cleave the auto‐inhibitory region from ROCK I. ROCK I can then facilitate actin remodelling and apoptotic body release via membrane blebbing. This pathway is highlighted in more detail in Fig. 1A. (B) Apoptotic caspase‐3 activity is implicated in the loading of CrkI into MVs, potentially via the cleavage of proteins within the focal adhesion complex, paxillin and cleaved p130^Cas^ [29]. (C) In non‐apoptotic scenarios, caspase‐2 cleaves ROCK II, removing its auto‐inhibitory region. ROCK II can then facilitate the actin remodelling required for MV release [30]. (D) Caspase‐3/‐8 are released via EVs as a means of regulating caspase activity in the cell, as an anti‐apoptotic mechanism [38, 39, 40, 41, 42]. (E) Non‐apoptotic caspase‐3 activity has been implicated in the cleavage of many EV‐associated proteins, including RBPs or ribosomal proteins. This process highlights caspases as potential regulators of EV RNA cargo [43]. (F) In cancer cells, caspase‐8 has been shown to cleave lysyl‐tRNA synthetase (KRS), making its PDZ domain accessible for Syntenin binding (a process involved in EV cargo loading). KRS is therefore loaded into MVBs and released via exosomes [44].

As highlighted previously, cleavage of ROCK I or ROCK II by caspases and granzymes respectively, induces blebbing of the plasma membrane [120, 121, 124] (Figs 1A and 2). Whilst this is often associated with AB release and cell death, caspase enzymes have also been shown to activate EV production in the absence of cell death (Fig. 2). Sapet et al. found that caspase‐2 is recruited by thrombin, even in the absence of cell death, with caspase‐2 activation required for ROCK II‐mediated EV release [30]. This study highlighted the potential involvement of non‐apoptotic caspase activity in the generation and release of EVs and indeed, non‐apoptotic caspase activity has been implicated in numerous aspects of EV biology. Recent deep profiling of caspase substrates revealed the caspase‐dependent cleavage of multiple Rho‐GTPases [152], which may also influence the cytoskeleton dynamics and EV biogenesis in different scenarios.

Aside from the involvement of caspases in ROCK‐mediated EV release, in 2013, Pallet et al. demonstrated that the caspase‐3‐dependent induction of apoptosis via serum starvation‐induced production of small EVs in a ROCK‐I independent manner [153]. These small EVs were distinct from ABs in their lack of nuclear components (DNA, histone H3, HMGB1), with inhibition of capase‐3 activity having a much greater effect on their release than on large ABs [127]. Whilst ABs were enriched for histones, ribosomal protein, ER and mitochondrial components, small apoEVs contain extracellular matrix and proteasomal proteins, alongside multiple autophagy components including lipidated LC3 [127, 153]. Of classical EV markers, these vesicles were positive for syntenin and annexins, but lacked detectable tetraspanins and TSG101. More recently, however, Leidal et al. also identified a population of EVs enriched for lipidated LC3, but also containing classical EV markers such as CD9, Alix and TSG101, alongside a strong enrichment for RNA binding proteins (RBPs) such as SAF‐B and HNRNPK, and again with histone proteins absent [98]. Although not considered in the study to be apoEVs, these cells were also cultured in serum‐free conditions, commonly used in the EV field to avoid contamination with bovine‐derived EVs. Secretion of these EVs was found to be dependent on ATG7 and ATG12, with their knockout having a significant impact on both EV protein and small RNA, snoRNA in particular. This secretion process was found to be largely ESCRT‐independent, but dependent on neutral sphingomyelinase and an interacting protein, Fan [98]. Since Fan is known to interact with caspase‐2 in a non‐apoptotic manner [154], these studies highlight the potential for involvement of multiple caspases in EV biogenesis and cargo determination in both apoptotic and non‐apoptotic scenarios.

## Caspase‐dependent regulation of EV content

### Caspases as EV cargo

Extracellular vesicles are important for the maintenance of homeostatic balance within cells [155]. One of the first proposed functions for EVs was to permit removal of transferrin receptor protein from reticulocytes, with later work showing a role in the removal of the disease‐causing alpha‐synuclein, beta‐amyloid and prion proteins (Parkinson's, Alzheimer's and Creutzfeldt‐Jakob disease respectively) [81, 82, 156, 157, 158]. However, in addition, they may act in the removal of lipid [159] and nucleic acids [160, 161, 162]. Furthermore, different research groups have suggested this mechanism could prevent the unintentional activation of endogenous cell signalling pathways, T‐cell activation and the DNA damage response [160, 162]. Consistent with this hypothesis, Böing et al. [38] have also shown that caspase‐3 can be packaged into EVs as a means of modulating intracellular levels of caspase activation. The release of these caspase‐3‐enriched EVs was independent of ROCK I, suggesting they result from an alternative pathway. Both caspase‐3 and caspase‐8 were also demonstrated to be released in EVs from serous salivary epithelial cells in mice [41]. This process had an anti‐apoptotic effect, protecting the epithelial cells from androgen deprivation‐induced cell death. Lastly, the biological relevance of EV‐mediated release of caspases was further demonstrated using human umbilical vein endothelial cells, in which inhibition of large EV release, and subsequent accumulation of caspase‐3, induced cell detachment [39, 40].

As previously described, the activity of caspase‐1 and the inflammasome protein complex are critical factors to mount effective innate immunological responses [133, 134]. According to Wang and collaborators, 19% of the caspase‐1 substrates and 5% of caspase‐1‐interacting proteins studied were extracellularly secreted, with only 28% of the caspase‐1 substrates present in the cytosol [45]. Moreover, 8 of the caspase‐1 substrates studied (RNH1, IL18, TBC1D15, SYAP1, AK2 TPI1, TPI1, HUWE1, CAP1) were previously associated with EVs [163, 164, 165, 166]. Caspase‐1, along with inflammasome proteins, have themselves been identified within EVs in other studies [31, 32, 33, 34]. Mitra et al. [32] determined that caspase‐1 release from activated immune cells acted to regulate apoptosis when taken up by recipient pulmonary microvascular endothelial cells during acute lung injury. Further evidence implicating this process in disease was derived by Wang et al., who determined that in coronary artery disease, whilst the upregulated caspase‐1 nuclear substrates were involved in pathways associated with cell death and chromatic remodelling, upregulated extracellularly secreted caspase‐1 substrates were associated with inflammation [45]. Caspase‐3 has also been shown to be loaded into EVs after hypoxia, along with caspase‐1, ‐8 and ‐12, via ROCK I pathways, as a potential means of transmitting ‘warning’ or pro‐inflammatory signals to the alveolar macrophages, facilitating neutrophil influx and subsequent lung inflammation [42]. Taken together, these studies suggest that EVs may facilitate the caspase‐mediated propagation of inflammation and/or cell death in neighbouring and remote cells, with these processes potentially dysregulated during disease states.

### Caspases as regulators of EV cargo loading

Several lines of evidence suggest that caspase activity could be instrumental in modulating EV cargo‐loading. In line with this, Kim and collaborators showed that caspase‐8 controls the EV‐mediated secretion of lysyl‐tRNA synthetase (KRS) [44]; an aminoacyl‐tRNA synthetase which regulates protein synthesis by loading amino acids to their cognate tRNAs [167]. KRS release from cancer cells is able to affect the inflammatory response [168]. Kim et al. [44] showed that the caspase‐8‐mediated cleavage of KRS facilitates its protein‐protein interaction with syntenin (a well‐characterised factor involved in EV cargo loading), which ultimately enables its inclusion into MVBs and subsequent release via exosomes. Intriguingly, KRS secretion was only observed under serum starvation conditions, and increased upon treatment with TNF‐α. Given that serum‐free culture conditions have been found to alter the protein composition of EVs [169], with serum‐free conditions known to result in caspase activation [129, 170], caspase activation may be more widely involved in determining EV cargo. Furthermore, activation of caspase‐8 under other cellular stress conditions could alter the content of EVs, and their effects on recipient cells, in a greater range of *in vivo* scenarios [171].

Non‐apoptotic caspase activity has been implicated in additional processes relating to cargo cleavage for EV incorporation. In a recent study by Weghorst et al., chick embryos were cultured *ex vivo* and treated with a caspase‐3 inhibitor or control solution [43]. Auditory brainstems from these chicks were harvested and proteomic analysis identified caspase‐3 substrates using the predicted caspase‐3 cleavage site ‘D/E ↓ X’ as reference (D and E representing aspartate and glutamate respectively, ↓ representing the cleavage site and X representing any amino acid except proline). Importantly, the proteome of the auditory brainstem was enriched for known caspase‐3 substrates unrelated to apoptosis, including gene ontology terms for EVs, ribosomal proteins, focal adhesions, chaperones and RNA‐binding proteins [43]. Furthermore, this study determined that caspase‐3 cleaves a large number of RBPs with recognised associations with EV biology (e.g. YBX‐1, SYNCRIP and hnRNPA2B1). Specifically, these proteins bind to RNAs via particular RNA recognition‐motifs, and subsequently modulate their incorporation into EVs [172, 173, 174, 175, 176]. RNA motifs, and their RBP associations, can have either cell retention or EV incorporation roles and therefore, caspases may enhance or diminish EV cargo loading depending on the cellular context [172, 177]. The enrichment of ribosomal proteins as caspase‐3 substrates also suggests caspase enzymes may be involved in the ribosomal RNA enrichment observed in EVs, particularly ABs, and warrants further investigation. The enrichment of focal adhesion proteins as caspase‐3 substrates provides additional support to the hypothesis made by Gupta et al. [29], who proposed that caspase‐mediated cleavage of focal adhesion proteins (paxillin or p130^cas^) was responsible for the liberation of Chicken tumour virus 10 regulator of kinase, CrkI, influencing its EV loading and release to modulate compensatory proliferation. Taken together, these studies highlight caspases as potential regulators of RNA and protein interactions, which may influence cargo stability and subsequent EV incorporation.

### Caspase‐mediated intraluminal EV cargo processing

**Fig. 3:**
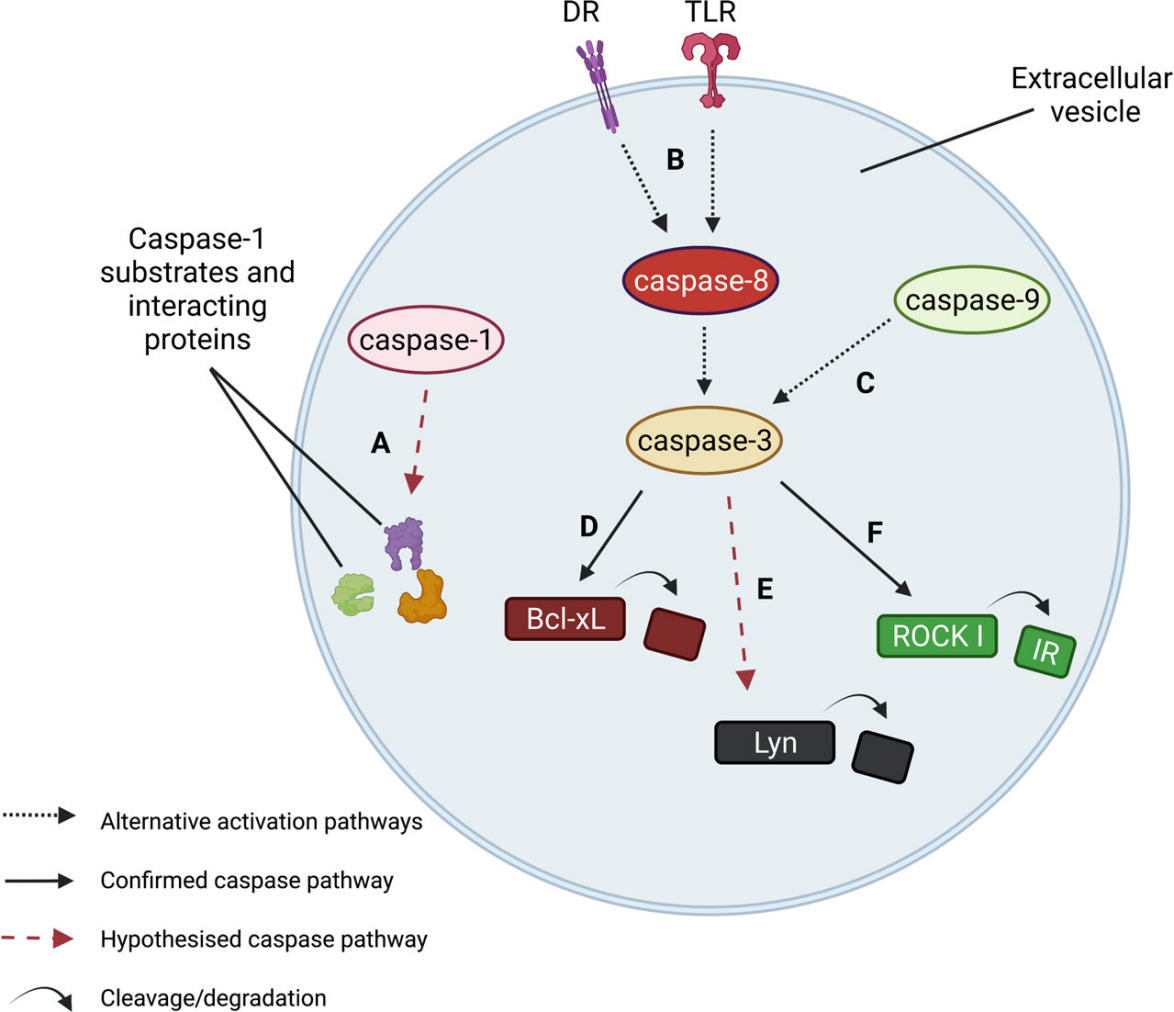
Examples of how caspase activity can alter cargo content within EVs. (A) Caspase‐1 has been found within EVs, along with caspase‐1 interacting proteins, suggesting the interaction between caspase‐1 and its interactors may take place within the EV, potentially facilitating intra‐vesicular caspase‐1 activity [31, 32, 33, 34, 45]. (B) Active caspase‐3 has been identified within EVs. Vardaki et al. reported the absence of cytochrome *c* or caspase‐9 within EVs, but the enrichment of the cleaved caspase‐8 along with the membrane DRs Fas and TLR4, and their adapter proteins Mydd88 and FADD, suggesting that extracellular stimuli may be responsible for the caspase‐3 activation observed within EVs, with this stimulation potentially occurring at the EV membrane surface [35]. Moon et al. also identified caspase‐3 within EVs, also alongside caspase‐1, ‐8 and ‐12 [42]. (C) Alternatively, Böing et al. [37] found caspase‐8 to be absent within EVs, whilst caspase‐9 was present and responsible for caspase‐3 activation. (D) Following on from pathway B, caspase‐3 cleavage of the abundant anti‐apoptotic Bcl‐x family member Bcl‐xL, facilitating the localisation of cleaved Bcl‐xL to the outer EV membrane, with this localisation necessary for uptake by malignant haematological cells, highlighting that caspase may act as an upstream determinant of EV uptake [35]. (E) Lyn, a Src tyrosine kinase, was selectively cleaved in EVs, with the subsequent relocation of Lyn from the EV membrane to the lumen. In cells, caspase‐3 is responsible for this cleavage. Active‐caspase‐3 was detected within EVs, suggesting intra‐vesicular Lyn cleavage by caspase‐3 may be occurring, with potential consequences on Src kinase regulation in uptake cells [36]. (F) Following on from pathway C, caspase‐3 activity was identified within EVs, with intra‐vesicular cleavage of ROCK I substrate confirmed [37].

In addition to the influence of caspase activity in the modulation of EV generation and loading, caspases have also been implicated in the process of EV cargo processing (Fig. 3). De Gassart et al. immunoprecipitated caspase‐3 from EVs, even when the parental cells were not undergoing cell death. EVs were then solubilised and caspase‐3 activity was assayed, confirming active caspase‐3 within EVs [36]. These authors also determined that Lyn, a Src tyrosine kinase, was selectively cleaved in EVs, with the subsequent relocation of Lyn from the membrane to the lumen. Cellular Lyn cleavage by caspase‐3 has been shown to result in its relocation from the membrane to the cytoplasm, with this release possibly resulting in increased kinase activity [178]. De Gassart et al. propose that similar caspase‐3‐mediated cleavage of Lyn may occur within EVs, which may have important consequences on EV‐mediated signalling to recipient cells, such as proliferation, differentiation and survival [36, 179].

Caspase activity within EVs was also demonstrated by Vardaki et al., with caspase‐3 cleaving the abundant anti‐apoptotic Bcl‐x family member Bcl‐xL, although not other Bcl‐x proteins [35]. Localisation of the cleaved Bcl‐xL to the outer EV membrane was found to be necessary for uptake by malignant haematological cells, raising the possibility that caspase activity may also act as an upstream determinant of EV uptake. Caspase activation can be triggered via two main pathways, via the intracellular stimuli, cytochrome *c* and caspase‐9 pathway, or via the extracellular stimuli and caspase‐8 pathway (Fig. 1A). Vardaki et al. reported the absence of cytochrome *c* or caspase‐9 within EVs, but the enrichment of the cleaved caspase‐8 along with the membrane receptors Fas and TLR4, and their adapter proteins myeloid differentiation primary response 88 (Mydd88) and Fas‐associated protein with death domain (FADD) [35]. This suggests that extracellular stimuli may be responsible for the caspase‐3 activation observed within EVs, with this stimulation potentially occurring at the EV membrane surface.

Böing et al. [37] also identified caspase‐3 activity within EVs when studying large platelet EVs, with intra‐vesicular cleavage of ROCK I substrate confirmed. Both procaspase‐3 and activated caspase‐3 were observed, with an increase in the activated form over 17 days of storage, indicating upstream caspase activity. Caspase‐3 can be cleaved by either caspase‐8 or caspase‐9 and, conversely to the results of Vardaki et al., Böing et al. [37] found caspase‐8 to be absent, whilst caspase‐9 was present and responsible for caspase‐3 activation. Testing the functional effects of these caspase‐3‐containing EVs in macrophages *in vitro*, however, revealed variable fractions of cells presenting signs of cell death (apoptosis or necrosis) which were not correlated with the number of EVs in the cell treatment. Non‐apoptotic consequences on recipient cells were not defined but would be an interesting avenue to explore in future work. Taken together, these studies suggest that, much like the cell‐specific nature of cellular caspase activity, caspase activity within EVs is a highly regulated and distinct process.

## Caspase‐dependent regulation of EV biological functions

Extracellular vesicle biology and caspase signalling have been intricately linked at various points along their signalling pathways. Gaining an improved understanding of these interactions has provided novel insights into key physiological processes, whilst also advancing our appreciation of pathological implications which can occur when these processes become disrupted (Table 1). Given that the caspase cascade has been highlighted as a therapeutic target for the treatment of conditions such as cancer, neurodegeneration, inflammation and sepsis [180, 181, 182], it is vital these complex interactions are fully understood to aid the safe and effective treatment of disease.

**Table 1:** Examples of caspase activity involvement in EV biology and the potential physiological or pathological implications of this interaction. ASC, apoptosis speck‐like protein containing a caspase recruitment domain; NLRP1, (NOD)‐like receptor protein‐1.

Caspase involvement in EV biology	Physiological/pathological implication	References
EV incorporation of caspase‐1 (along with caspase‐1 interacting proteins)	EV‐loaded active inflammasome complexes propagate inflammation via active caspase‐1. In coronary artery disease, caspase‐1 substrates upregulated, with extracellularly secreted caspase‐1 substrates associated with inflammation.	[45]
	Caspase‐1 and inflammasome components are enriched in cerebrospinal fluid EVs from patients with poor outcome after traumatic brain or spinal cord injury (SCI). EV‐mediated delivery of ASC (an inflammasome component) siRNA decreased inflammasome/caspase‐1 activation in rodents following SCI, highlighting this as potential therapeutic target.	[31]
	Active caspase‐1 (and ASC) shed via EVs from stimulated monocytes, inducing cell death in recipient cells. EV‐loaded caspase‐1 observed during sepsis, as a potential apoptotic signalling factor (and therapeutic target).	[33, 34]
	Caspase‐1 release from activated immune cells regulated apoptosis in recipient pulmonary microvascular endothelial cells during acute lung injury.	[32]
EV incorporation of caspase‐3	Intra‐vesicular cleavage of Bcl‐xL by caspase‐3 required for EV uptake by malignant blood cells. EV‐loaded caspase‐8 and membrane receptors suggest caspase‐3 activation may occur within EVs, potentially via EV membrane stimulus. Caspase‐3 inhibition resulted in reduced cell proliferation in recipient tumour cells.	[35]
	Caspase‐9 is present and responsible for caspase‐3 activation within EVs. Caspase‐loaded EVs did not induce apoptosis in recipient cells. Caspase‐3 release via EVs modulated intracellular caspase activity levels, preventing cell death.	[37, 38, 39, 40]
	Treatment of cells with caspase‐3 inhibitor increased the molecular weight of EV‐loaded Lyn (Src tyrosine kinase). Caspase‐3 cleavage of Lyn within EVs has potential signalling function in recipient cells related to proliferation, differentiation, motility and survival.	[36]
	Caspase‐3 (and caspase‐1, ‐8, ‐12) EV‐loaded after hypoxia via ROCK I pathways, as a potential means of transmitting ‘warning’ or pro‐inflammatory signals to alveolar macrophages, facilitating neutrophil influx and lung inflammation.	[42]
EV incorporation of caspase‐8	The release of caspase‐8 (and caspase‐3) via EVs from serous salivary epithelial cells in mice was an anti‐apoptotic mechanism, controlling intracellular caspase levels.	[41]
Caspase enzymes as potential modulators of EV cargo	Non‐apoptotic caspase‐3 cleavage of EV‐associated proteins, RBPs and ribosomal proteins, potentially regulating EV cargo loading, particularly RNA. This has potential effects on signalling and gene expression in recipient cells.	[43]
	Lysyl‐tRNA synthetase (KRS) is released from cancer cells to modulate the inflammatory response, with caspase‐8 cleavage of KRS exposing the KRS PDZ syntenin‐binding domain, enhancing its EV‐loading.	[44]
	Pan‐caspase inhibitor (Z‐VAD) reduced production of CrkI‐loaded EVs. Caspase activity hypothesised to be involved in breakdown of the focal adhesion complex to aid liberation of CrkI and EV‐loading. CrkI‐loaded EVs activated proliferation in neighbouring cells via the JNK pathway.	[29]
Caspase enzymes modulate membrane blebbing	ROCK‐I cleavage by caspase‐3, ‐7 or‐2 results in membrane blebbing to facilitate the formation of ABs. Controlled cell breakdown via ABs allows immune cells to clear cell debris. ABs also trigger signalling events (TLR activation, suppression of proliferation and pro or anti‐apoptotic functions) in recipient cells.	[120, 121, 122, 125, 127, 128, 129, 130, 196]
	Caspase‐2 recruited by thrombin, even in the absence of cell death, with caspase‐2 activation required for ROCK II‐mediated EV release. This highlights non‐apoptotic caspase activity in the generation and release of EVs.	[30]

Whilst uncovering potential mechanistic insights to EV biogenesis through the identification of caspase‐3 cleavage in auditory brainstem EVs, the work of Weghorst et al. also highlighted the implications of these EVs in a non‐apoptotic, neurodevelopmental, context. The authors identified two caspase‐3 substrates, neuronal adhesion proteins NCAM and Ng‐CAM (homologous to mammalian L1CAM) with known roles in axon guidance [43]. Thus cleavage of EV proteins is proposed as a novel mechanism by which caspase‐3 may affect auditory brainstem development.

The potential pathological consequences of caspase‐modulated EV cargo loading and processing have been highlighted by a number of studies. The work of Kim et al. demonstrated KRS is cleaved by caspase‐8 within cancer cells to facilitate its release via exosomes and, since KRS release from cancer cells is known to affect the inflammatory response, this modulation is relevant in the building of a niche with the potential to promote cancer cell survival and proliferation [44, 168, 183]. Vardaki et al. [35] have demonstrated decreased tumour cell proliferation in EV‐recipient cells as a result of caspase‐3 inhibition, likely via an effect on EV uptake, highlighting its important involvement in cancer progression and potential as a target for therapeutic intervention.

The process of cell proliferation and tissue regeneration is an area of extensive overlap between caspase activity and EV function. As highlighted previously, EVs and caspases were together implicated in the process of compensatory proliferation by Gupta et al., with these authors describing ‘apoptotic compensatory proliferation signalling vesicles’ or ACPSVs [29, 184]. These CrkI‐containing MVs from HeLa cells were relatively large (1.76 ± 1.04 µm diameter), with only 3–5 released per cell by a small subpopulation (5%) of cells. These EVs were shown to activate proliferation in neighbouring cells via the JNK pathway. Involvement of the caspase pathway was determined via the pre‐treatment of cells with a pan‐caspase inhibitor (Z‐VAD), which significantly reduced both ACPSV formation and compensatory proliferation.

Hervera et al. have also suggested a role for EVs in immune‐mediated repair and regeneration, involving reactive oxygen species (ROS) signalling [185]. These authors determined that EVs containing functional NADPH oxidase 2 complexes (a source of ROS), were released from macrophages and incorporated into injured axons via endocytosis to promote regeneration [185, 186]. Release of these EVs was found to be dependent on an inflammatory response involving the Fractalkine chemokine receptor, CX3CR1. Expression of CX3CL1 (Fractalkine) was previously shown to be highly down‐modulated by inhibitors of calpain (modulators of caspase activity) and significantly decreased with caspase‐1, ‐3, or ‐9 inhibition in human umbilical vein endothelial cells [187]. Interestingly, Fractalkine was also shown to be enriched in EVs derived from endothelial cells stimulated with the inflammatory cytokine, TNF‐α [188]. Caspases have been heavily implicated in the process of apoptosis‐induced proliferation in which the secretion of mitogens by apoptotic cells promotes the proliferation of nearby cells, including via extracellular ROS release [189, 190, 191]. Taken together, these studies highlight that caspase enzymes may be well positioned within the cell to mediate the bi‐directional EV‐mediated signalling involved in the inflammatory response and tissue regeneration.

## Conclusion

Caspase enzymes, originally considered as primarily drivers of programmed cell death, are now being revealed as intricately associated with many aspects of cell function and multicellular life, from differentiation and development to stress response and cell communication. Whilst caspase activity is known to contribute to the membrane blebbing required for apoptotic body formation, their role in EV biology expands beyond this function, in EV release, cargo sorting and processing, as well as EV uptake. Given that a baseline level of caspase activity maintains vital cellular functions beyond apoptosis, caspases are potentially involved in the generation of a spectrum of EVs in both healthy and stressed cells. This blurs the lines between apoptotic and ‘normal’ EVs, with specific environmental conditions likely resulting in a unique mixture of EVs. Greater consideration is therefore needed when assessing potential cellular stresses, such as serum starvation, when studying EV biology.

Whilst caspases are known to be involved in various aspects of EV biology, more work is needed to define the molecular mechanisms of these processes, particularly under stress or in disease states. Improved tools, especially *in vivo* models, are needed to explore the fundamental mechanisms of EV biogenesis and transfer in complex multicellular environments, in both physiological and pathological scenarios [192, 193, 194, 195]. Given that caspase enzymes are often the target of therapeutic molecules, the involvement of these enzymes in long‐range EV‐mediated cell communication mechanisms highlights potential non‐autonomous effects of manipulating their activities. A better understanding of the interplay between caspases and EV biology might lead towards new therapeutic opportunities for diseases, whilst minimising their off‐target consequences.

## Conflict of interest

The authors declare no conflicts of interest.

## Author contributions

CH and ERD contributed equally to researching and writing of the article. CH produced figures. LAB‐L provided critical intellectual and editorial input. All authors reviewed the final article before submission.

## Data Availability

This manuscript does not contain original data or unpublished information. All of the information described has been previously included in the references cited in the manuscript and therefore can be found there.
